# Hospitalizations in Brazil according to National Health Survey estimates, 2013 and 2019

**DOI:** 10.11606/s1518-8787.2023057004395

**Published:** 2023-09-29

**Authors:** André Oliveira Andrade, Sandra Rêgo de Jesus, Sostenes Mistro

**Affiliations:** I Universidade Federal da Bahia Instituto Multidisciplinar em Saúde Programa de Pós-Graduação em Saúde Coletiva Vitória da Conquista BA Brazil Universidade Federal da Bahia. Instituto Multidisciplinar em Saúde. Programa de Pós-Graduação em Saúde Coletiva. Vitória da Conquista, BA, Brazil; II Universidade Estadual do Sudoeste da Bahia Departamento de Ciências da Saúde Vitória da Conquista BA Brazil Universidade Estadual do Sudoeste da Bahia. Departamento de Ciências da Saúde. Vitória da Conquista, BA, Brazil; III Universidade Federal da Bahia Instituto Multidisciplinar em Saúde Vitória da Conquista BA Brazil Universidade Federal da Bahia. Instituto Multidisciplinar em Saúde. Vitória da Conquista, BA, Brazil

**Keywords:** Hospitalization, Hospitals, Hospital Statistics, Epidemiological Surveys, Cross-sectional Studies

## Abstract

**OBJECTIVE:**

To compare the profile and prevalence of hospitalizations in Brazil based on estimates from the National Health Survey (PNS), 2013 and 2019.

**METHODS:**

A cross-sectional study that used data from the 2013 PNS and the 2019 PNS. The outcome was having been hospitalized for 24 hours or more in the last 12 months. We calculated the proportion of the population in different categories of age group, presence or absence of chronic diseases, and perception of health status. We estimated the total number of hospitalizations and the proportion corresponding to each category of age group, chronic disease, and perceived health status. We calculated the prevalence of hospitalization according to geographic, socioeconomic, and health conditions. We compared the estimates of two editions of the PNS using Student’s t-test for independent samples. We considered significant differences when the p-value was less than 0.01. And finally, we compared hospitalization estimates with administrative data to assess data consistency.

**RESULTS:**

We observed that the proportion of chronically ill people in the population increased from 15.04% to 31.48%. This group was responsible for 36.76% of the total number of hospitalizations in 2013 and 57.61% in 2019. The prevalence of hospitalizations increased significantly between the two surveys and the increases were higher in the Southeast region and among people who have private health insurance. A discrepancy was found between administrative data and survey estimates. Obstetric hospitalizations and health insurance hospitalizations were underestimated.

**CONCLUSION:**

There was an increase in overall hospitalization rates in the period between the PNS 2013 and PNS 2019, especially among people with better access to health services. The hospitalization profile also changed—in the 2013 PNS, hospitalizations of people without chronic diseases predominated. This was reversed in PNS 2019.

## INTRODUCTION

The hospital is the most expensive piece of equipment in a health system and can represent a significant source of wasted resources, to the point of compromising the efficiency and effectiveness of this system^1,2^. In 2019, spending on hospital and outpatient care accounted for 49.94% of public health expenditures in Brazil, more than double what was spent on primary care in the same year^3^. However, despite this large amount of resources, necessary to maintain hospital care, one cannot imagine doing without the effectiveness provided by the technological apparatus concentrated in a hospital, especially in critical health conditions.

It is likely that the spending on hospital care in Brazil would have been proportionally higher without the expansion of investments in primary care that occurred after the creation of the Sistema Único de Saúde (Unified Health System – SUS) and, mainly, after the implementation of the Family Health Strategy. From these changes, there was a significant improvement in health care. The majority place of care migrated from the outpatient clinic to the health post/center^4^and, gradually, the hospital has ceased to be the gateway to the health system. However, in 2019, more than 25% of people still sought the hospital as a place of first health care^5^.

Hospital and primary care complement each other within a health system. Primary care has the function of comprehensive and longitudinal care for the individual’s health. Within its scope, preventive and educational actions are promoted, to rehabilitation or even palliative care^6^. In turn, the hospital must resolve certain complex situations, preferably infrequently. Therefore, the aim is a health system with robust and effective primary care, which provides the lowest possible hospitalization rates^7^.

Hospitalization rates can be influenced by social, economic, behavioral, environmental, and demographic factors, the same factors that influence a population’s health^8^. Conditions such as unemployment, low income, restrictions and lack of family involvement are, for example, predictors of a higher frequency of children’s hospitalization^9^. In the United States, 11% of the variability in hospitalization due to influenza is determined by social factors^10^. Groups that have worse socioeconomic indicators, such as low income and education, are 2.4 times more likely to be hospitalized or have more severe respiratory diseases^11^. The supply of health services is another factor that strongly affects hospitalization rates. Castro et al.^12^showed in 2005 that “the greater the average number of beds per population, the greater the chance of hospitalization, and the greater the number of physicians per population, the lower the chance of hospitalization” (our translation). Likewise, there are other factors associated with hospitalizations, albeit weakly, such as the percentage of illiteracy, the proportion of SUS beds, urbanization, and coverage by health insurance plans^13^.

Although hospitalization rates are influenced by several variables, in different dimensions, the main factor associated with hospital admissions is the need for health. People who are hospitalized have a higher number of chronic diseases, worse health status, worse functional status, and more restrictions on carrying out usual activities^14^. Likewise, the prevalence of hospitalization in people with multimorbidities is double that of people without multimorbidities^15^. These relationships between chronic diseases, comorbidities, and hospitalizations should produce important changes in hospitalizations in Brazil, since the country is undergoing a demographic and epidemiological transition with an increase in chronic diseases^16^.

The population’s demographic and epidemiological profile influences the demand for hospitalization. In Brazil, since 1980, fertility and birth rates have decreased while life expectancy at birth has increased^17^. With fewer births, the demand for obstetrics and pediatrics beds has been decreasing and naturally there has been a reduction in supply. However, the prevalence of chronic diseases increases with an aging population. Paradoxically, this reduction in the supply of beds and decrease in the hospitalization rate has also been observed in medical and surgical practice, although to a lesser extent^17^.

In 2013, the Brazilian Institute of Geography and Statistics (IBGE) in association with the Ministry of Health (MS) carried out the first *Pesquisa Nacional de Saúde* (PNS – National Health Survey). The research had many objectives, which included evaluating the national health system in terms of access to and use of its services, as well as measuring access to medical care at different levels of care^18^. The survey revealed that 53.4% of households were registered in Family Health Units, which correspond to Primary Care in Brazil^19^. Later, in 2019, the second edition of the survey was carried out, with changes in some questions and an expansion of the questionnaire^20^. Both editions of the PNS presented information on hospitalizations and, in this second edition, an increase in the registration of households in Primary Care was observed to 60.0%^21^.

According to PNS 2013 data, 6.0% of individuals had been hospitalized for 24 hours or more in the last 12 months^19^, a prevalence that increased to 6.6% in PNS 2019^21^. From 1999 to 2016, the rate of hospitalizations financed by the SUS had been gradually decreasing^15,17,22^. After this sustained reduction, in 2017 an increase in this rate was identified as compared to the previous year^22^, and this increase was also noticed in the last edition of the PNS, in 2019. It is still unclear whether these facts mean points outside the curve or a new growth trend of hospitalizations in Brazil. Our study intended to clarify this issue. Based on the 2013 and 2019 PNS estimates, we aimed to compare the prevalence of hospitalizations according to geographic, socioeconomic and health conditions and to identify any changes in the hospitalizations profile in Brazil.

## METHODS

This work is a serial cross-sectional study that used data from the PNS 2013 and PNS 2019, which took place respectively between August 2013 and February 2014^23^, and between August 2019 and March 2020^20^, whose object of study was the Brazilian population.

The 2013 PNS interviews were conducted in 64,348 households, in approximately 1,600 municipalities throughout Brazil^24^, with information on 205,546 residents, referring to a population projection of 199,551,444 inhabitants. The PNS 2019 interviews were conducted in 94,114 households, referring to 279,382 residents and a population projection of 209,589,607 inhabitants. The two surveys followed a similar methodology. The items were divided into 3 groups. The first provided information about the household and visits by family health teams or endemic agents. The second investigated the general characteristics of each resident, income, health insurance coverage, use of health services, and health status of individuals. Questions in the third group were outside the scope of this study and were not used. The questionnaire, the interviewer’s manual and the microdata are available from the IBGE website^25^.

In this study, we used the following condition as the outcome: having been hospitalized for 24 hours or more in the last 12 months. As a health need is the main determinant of hospital use, we describe the participation of chronic illness and perception of poor health status in the Brazilian population and in total hospitalizations. The age group was also included in the description. From the microdata from the two PNS, we estimated the proportion of the population in different categories of age, presence or absence of chronic diseases and perception of health status, and the number of hospitalizations related to each category. With the estimated mean total numbers, and their respective confidence intervals, we calculated how much each category of these variables represented, proportionally, in the general total of hospitalizations, in eachPNS edition.

To analyze changes in the prevalence of hospitalization, we used the factors described in Andersen’s behavioral model of health service use^30^. We used the following variables: as predisposing factors – sex, age group, skin color, and level of education; as facilitating factors – urban or rural housing, usual place of care, presence or absence of medical health insurance, household income *per capita*, and region of the federation; and as health need – perception of health and presence or absence of chronic disease. In the age group variable, ages were grouped into 6 categories: 0–5 years, 6–17 years, 18–29 years, 30–39 years, 40–59 years, and 60 years of age or older. All variables are categorical. The prevalence of hospitalization and respective 95% confidence intervals were calculated according to the mentioned factors. In the prevalence calculations, hospitalizations for childbirth were excluded.

The estimates found in the two editions of the PNS were compared using Student’s t-test for independent samples. We considered significant differences when *p* was less than 0.01^31^.

The consistency of survey data was assessed by comparing administrative data with estimates from the two editions. Hospitalizations estimated by the PNS were broken down into hospitalizations covered by the SUS, hospitalizations covered by private health insurance, and obstetric hospitalizations. Information on births in hospitals in 2013 and 2019 was obtained from the website IBGE – Civil Registry Statistics, through the IBGE Automatic Recovery System (SIDRA)^32^. Hospitalizations through the SUS in 2013 and 2019 were obtained from the SUS Hospital Information System (SIH/SUS)^33^. Information on hospitalizations covered partially or fully by private health insurance was obtained from the website of the Agência Nacional de Saúde Suplementar (National Agency of Supplementary Health – ANS)^34^, and referred to the years 2014 and 2019. Data referring to the year 2013 were not found.

As this is a study that used complex sampling, we used the Stata® statistical software, version SE 15.1 for data analysis, which, through its survey module, takes into account the effect of the sampling plan. In the adjustment, strata, primary sampling units and sample weights were incorporated.

PNS 2013 was approved by opinion no. 328.159 of the National Research Ethics Committee. PNS 2019 was approved by opinion no. 3,529,376 of the National Research Ethics Commission. All participants signed, in both editions, an informed consent form, assuring them anonymity and the possibility of withdrawing from the study at any time.

## RESULTS

According to the two surveys’ estimates, there were changes in the demographic and epidemiological profile of the Brazilian population between 2013 and 2019. A significant change was observed among people aged 6 to 29 years and among people aged 40 years or older. The percentage of people with chronic diseases increased significantly between the two PNS editions, and the perception of health status changed in 4 of the 5 categories, with an increase in the very good, fair, and very poor categories. This information is detailed in Table 1.

**Table 1:** Proportional distribution (%) of the population in Brazil, according to age group, presence of chronic disease, and perception of health status, according to estimates from PNS 2013 and PNS 2019.

Characteristic	PNS 2013 – Percentage of population (95%CI)	PNS 2019 - Percentage of population (95%CI)
Age range (years)		
0–5	7.68 (7.49–7.87)	7.44 (7.26–7.63)
6–17*	19.37 (19.07–19.68)	16.61 (16.38–16.85)
18–29*	19.36 (19.06–19.67)	17.32 (17.05–17.60)
30–39	15.44 (15.15–15.73)	15.40 (15.14–15.67)
40–59*	24.98 (24.66–25.31)	26.81 (26.51–27.11)
≥ 60	13.17 (12.83–13.52)	16.41 (16.04–16.79)
Chronic disease		
Present*	15.04 (14.64–15.44)	31.48 (31.09–31.88)
Absent*	84.96 (84.56–85.36)	68.52 (68.12–68.91)
Perception of health status		
Very good*	14.40 (13.84–14.97)	17.90 (17.39–18.42)
Good*	59.69 (59.11–60.28)	55.10 (54.60–55.59)
Fair*	21.56 (21.14–21.99)	22.34 (21.99–22.70)
Poor	3.59 (3.43–3.75)	3.72 (3.60–3.85)
Very poor*	0.75 (0.70–0.82)	0.94 (0.86–1.02)

95%CI: 95% confidence interval; PNS: National Health Survey.

* *p*-value < 0.01 in Student’s t-test for comparison of independent samples.

Changes in the hospitalization profile were also observed. While hospitalizations by people without chronic diseases predominated in the 2013 PNS, patients with chronic diseases predominated in the 2019 PNS, as shown in Table 2.

**Table 2:** Proportional participation (%) in total hospital admissions, by age group, by presence of chronic disease, and by perception of health status, according to estimates from PNS 2013 and PNS 2019.

Characteristic	PNS 2013 – Percentage of total hospitalizations (95%CI)	PNS 2019 - Percentage of total hospitalizations (95%CI)
Age range (years)		
0–5	9.59 (8.42 -10.77)	8.87 (7.80–9.95)
6–17*	9.96 (8.64–11.29)	6.70 (5.97–7.44)
18–29	15.62 (14.09–17.15)	13.86 (12.69–15.02)
30–39	15.17 (13.03–17.30)	14.75 (13.40–16.09)
40–59	24.09 (21.97–26.21)	27.47 (25.51–29.43)
≥ 60	25.56 (22.21 - 28.91)	28.35 (26.42–30.28)
Chronic disease		
Present*	36.76 (33.33–40.19)	57.61 (54.36–60.86)
Absent*	63.24 (58.36–68.12)	42.39 (39.91–44.86)
Perception of health status		
Very good	7.73 (6.79–8.67)	9.28 (8.44–10.11)
Good	40.07 (36.06 -44.07)	36.34 (34.13–38.56)
Fair	33.12 (30.34 -35.90)	34.47 (32.29–36.66)
Poor	12.80 (11.30 -14.30)	13.94 (12.44–15.44)
Very poor	6.28 (4.54 -8.02)	5.97 (5.04–6.90)

95%CI: 95% confidence interval; PNS: National Health Survey.

*p-value < 0.01 in Student’s t-test for comparison of independent samples.

Excluding hospitalizations for childbirth, the prevalence of hospitalization increased significantly from 5.32% in the 2013 PNS to 5.81% in the 2019 PNS. There was a statistically significant increase in prevalence between 2013 and 2019 in the Southeast Region in both sexes, in people with white skin color, in people with no schooling, in people with complete high school education, in people living in urban areas, in people with health insurance, in people who always go the same place when they need health care, and for people with a household income *per capita* greater than 3 and up to 5 minimum wages. On the other hand, significant reductions in prevalence were observed in people with chronic disease and in people without chronic disease, as can be seen in Table 3.

**Table 3:** Proportion of people (%) hospitalized in the last 12 months, excluding obstetric admissions. National Health Survey, Brazil, 2013 and 2019.

characteristic	PNS 2013 n = 203,883	PNS 2019 n = 277,195
	
	% (95%CI)	% (95%CI)
General*	5.32 (5.13–5.52)	5.81 (5.64–5.98)
Region		
North	5.08 (4.67–5.52)	4.68 (4.43–4.94)
Northeast	4.92 (4.68–5.16)	5.06 (4.84–5.29)
Southeast*	4.95 (4.58–5.36)	6.10 (5.77–6.45)
South	6.68 (6.24–7.15)	6.47 (6.13–6.83)
Midwest	6.53 (6.10–6.99)	6.88 (6.46–7.34)
Sex		
Male*	4.95 (4.71–5.19)	5.44 (5.23–5.66)
Female*	5.67 (5.42–5.93)	6.15 (5.93–6.39)
Age range (years)		
0–5	7.87 (7.20–8.60)	8.32 (7.49–9.24)
6–17	2.83 (2.56–3.12)	2.63 (2.42–2.86)
18–29	3.33 (3.03–3.66)	3.47 (3.21–3.74)
30–39	4.44 (4.07–4.84)	4.61 (4.28–4.96)
40–59	5.91 (5.56–6.29)	6.31 (6.01–6.64)
≥ 60	10.23 (9.58–10.92)	10.58 (10.13–11.05)
Skin color		
White*	5.59 (5.33–5.86)	6.19 (5.94–6.44)
Black	4.78 (4.28–5.34)	5.38 (4.97–5.83)
Yellow	5.48 (3.94–7.59)	4.92 (3.59–6.72)
Brown	5.13 (4.85–5.43)	5.56 (5.28–5.85)
Indigenous	6.33 (4.39 - 9.03)	5.55 (3.91–7.83)
Education level^a^		
No schooling*	6.83 (6.29–7.42)	8.00 (7.37–8.68)
Incomplete primary education	5.29 (5.01–5.60)	5.65 (5.40–5.92)
Primary education	4.54 (4.05–5.07)	5.31 (4.84–5.84)
Incomplete secondary education	3.97 (3.39–4.65)	4.08 (3.66–4.55)
Secondary education*	4.42 (4.09–4.77)	5.23 (4.93–5.55)
Incomplete higher education	4.07 (3.32–4.97)	4.92 (4.31–5.62)
Higher education	5.51 (4.96–6.11)	5.86 (5.45–6.31)
Place of residence		
Urban*	5.39 (5.17–5.61)	5.91 (5.72–6.11)
Rural	4.91 (4.56–5.29)	5.20 (4.91–5.51)
Health perception		
Very good	3.10 (2.76–3.47)	3.28 (3.00–3.57)
Good	3.77 (3.54–4.01)	4.12 (3.92–4.33)
Fair	8.39 (7.98–8.82)	9.07 (8.71–9.44)
Poor	16.60 (15.34–17.96)	18.02 (16.72–19.41)
Very poor	27.68 (24.43–31.18)	26.66 (24.05–29.45)
Health Insurance^b^		
People with health insurance*	6.45 (6.10–6.81)	7.60 (7.22–7.99)
People without health insurance	4.88 (4.65–5.12)	5.18 (5.02–5.35)
Chronic disease		
People with chronic disease*	12.20 (11.61–12.82)	10.59 (10.23–10.96)
People without chronic disease*	4.09 (3.91–4.28)	3.61 (3.46–3.76)
Place you go when you need health care		
Always the same place*	5.57 (5.34–5.81)	6.11 (5.92–6.31)
Search different locations	4.45 (4.16–4.76)	4.82 (4.56–5.10)
Household income *per capita*^c^		
Up to ¼ minimum wage	5.00 (4.51–5.54)	4.91 (4.55–5.30)
More than ¼ to ½ minimum wage	4.71 (4.38–5.05)	4.80 (4.49–5.12)
More than ½ up to 1 minimum wage	5.41 (5.01–5.85)	6.02 (5.72–6.34)
More than 1 to 2 minimum wages	5.41 (5.08–5.75)	5.94 (5.63–6.27)
More than 2 to 3 minimum wages	5.73 (5.17–6.34)	6.11 (5.51–6.76)
More than 3 to 5 minimum wages*	5.16 (4.46–5.97)	6.96 (6.28–7.71)
More than 5 minimum wages	6.54 (5.79–7.38)	7.38 (6.65–8.18)

95%CI: 95% confidence interval; PNS: National Health Survey.

* *p* value < 0.01 in Student’s *t*-test for comparing independent samples.

^a^ In the variable degree of education, n was 189,461 in PNS 2013 and 259,408 in PNS 2019.

^b^ There was a change in this item: in the 2013 PNS the question was whether you had any health insurance, medical or dental; in PNS 2019 this item was expanded, asking about the presence of a medical health insurance.

^c^ In the household income *per capita* variable, n was 277,023 in the 2019 PNS.

When analyzing admissions for childbirth, estimated in each survey, with administrative data, referring to live births in equivalents years, we noticed different values. Similarly, we noticed a difference in non-obstetric hospitalizations, both in those financed by the SUS and in those financed by health insurance. The values are shown in Table 4.

**Table 4:** Comparison between administrative data and the number of hospitalizations according to the PNS 2013 and PNS 2019 estimates

Type of hospitalization	PNS 2013 estimate (95%CI)	2013 - Administrative data	PNS 2019 estimate (95%CI)	2019 - Administrative data
Obstetric admissions:				
SUS	1,247,725 (1,148,557–1,346,893)	2,113,466^a^	1,341,126 (1,242,042–1,440,211)	2,225,147^a^
Probably SUS	7,323		1,498	
Health insurance	425,999 (354,489–497,508)	713,840^b^	457,670 (399,004–516,336)	648,174^c^
Paid (and without health insurance)	83,760 (48,100–119,420)		88,491 (63,307–113,674)	
Total	1,781,091 (1,662,829–1,899,354)	2,771,629^d^	1,908,288 (1,798,038–2,018,539)	2,752,386^d^
Non-obstetric hospitalizations				
SUS	10,631,713 (9,989,486–11,273,940)	9,407,371^a^	11,481,234 (11,005,629– 11,956,839)	10,128,465^a^
Probably SUS	91,687 (39,810–143,565)		30,294 (–)	
Health insurance	4,239,450 (3,765,530–4,713,370)	6,084,261^b^	4,964,168 (4,674,907–5,253,428)	6,899,526^c^
Paid (and without health insurance)	682,632 (591,637–773,627)		754,818 (677,270–832,367)	
Total	15,939,768 (15,083,996–16,795,540)		17,409,054 (16,784,781–18,033,327)	
Total hospitalizations				
SUS	11,879,438 (11,210,696–12,548,180)	11,520,837^a^	12,822,361 (12,315,955–13,328,767)	12,353,612^a^
Probably SUS	99,011 (47,512–150,509)		31,791 (–)	
Health insurance	4,665,449 (4,176,951–5,153,946)	6,798,101^b^	5,421,838 (5,111,183–5,732,493)	7,547,700^c^
Paid (and without health insurance)	766,391 (672,239–860,545)		843,309 (765,342–921,276)	
Total hospitalizations	17,720,859 (16,847,839–18,593,879)		19,317,343 (18,657,337–19,977,349)	

95%CI: 95% confidence interval; PNS: National Health Survey; SUS: Sistema Único de Saúde (Unified Health System); SIH/SUS: Hospital Information System; ANS: Agência Nacional de Saúde Suplementar (National Agency of Supplementary Health); IBGE: Instituto Brasileiro de Geografia e Estatística.

^a^ Hospital Production SIH/SUS).

^b^ ANS – Assistance data for the sector for 2014.

^c^ ANS – Assistance data for the sector for 2019.

^d^ IBGE, Civil Registry Statistics – 2,802,849 live births in hospitals in 2013 and 2,783,409 live births in hospitals in 2019; considering twin pregnancies, these numbers are equivalent to 2,771,629 and 2,752,386 births, respectively.

## DISCUSSION

Our study shows an increase in the estimated prevalence of hospitalizations between the 2013 and 2019 PNS editions. This growth was accompanied by important changes in the population’s epidemiological and demographic profile, especially in the doubling of the percentage of people with chronic diseases, along with a decrease in the percentage of those aged 6 to 29 and an increase among those aged 40 and over.

Similarly, other studies, with data from the 2013 and 2019 PNS, show that the prevalence of chronic diseases has increased in Brazil^35,36^. The concomitant increase observed in the proportion of people with chronic diseases and people in middle age explains an apparently paradoxical finding. As can be seen in our results, there was a reduction in the prevalence of hospitalizations in people with chronic diseases. With a greater number of people in the middle age group, there are more individuals with chronic diseases, but who have not yet had complications. Consequently, there has been an increase in the proportion of people with chronic diseases who do not require hospitalization.

Although the prevalence of hospitalization has decreased among people with chronic diseases, as they have proportionally increased, and hospitalization among them remains very high, there has been an increase in the general prevalence of the population. The participation of patients with chronic diseases in total hospitalizations increased from 36.76% to 57.61% between the two PNS editions. These findings demonstrate the influence of the epidemiological profile and demography on hospitalization rates in Brazil.

In Brazil, the epidemiological transition is characterized by a triple burden of disease. Non-transmissible chronic diseases (NTCDs) coexist with a high incidence and prevalence of infectious and parasitic diseases (IPD) and external causes^37^. In 2019, proportionally, the burden of IPD was higher in early childhood, the burden of external causes was higher in young male adults, and the burden of NTCDs increased with age^37^. According to PNS 2019 estimates, in more than 40% of hospitalizations, there was no diagnosis of chronic disease. If hospitalizations reflect the burden of disease, we will have a predominance of hospitalizations due to IPD, in early childhood, due to external causes, in young males, and due to obstetric causes in young females.

Emerging situations also influence hospitalizations. In 2020, due to the covid-19 pandemic, there was a 15% decrease in the hospital admission rate, accompanied by a 9% increase in in-hospital lethality of patients with cardiovascular diseases admitted through the SUS^38^.

A study on the profile of hospital admissions by the SUS between 2013 and 2017 showed that in 24.4% of hospitalizations, the patient was 60 years old or over^39^. We estimate that people aged 60 or over accounted for 25.56% of all hospitalizations in the 2013 PNS and 28.35% in the 2019 PNS, but without a statistically significant difference between the two surveys. Brazil has been increasing life expectancy due to reductions in child mortality and cardiovascular disease mortality, despite the negative influence of mortality from external causes, which occurs mainly in young male adults^40^. The increase in life expectancy is due, in part, to the improvement in health care. The challenge is not only to increase life expectancy, but mainly years with quality of life^41^.

Similarly, we found that people with poor or very poor health status correspond to less than 5% of the population, but account for almost 20% of all hospitalizations. These people probably have worse health, and we already know that people in this situation are the ones who need hospitals the most^14^. Adequate primary care and an integrated health care network can reduce hospitalizations, reduce these people’s hospital stay, and also reduce readmissions^42^.

In Brazil, between 2013 and 2019, the proportion of individuals who consulted a doctor in the last year increased. This increase was greater among SUS users than among health insurance holders^36^. This is probably a reflection of the primary care expansion policy, with the Family Health Strategy.

An adequate supply of physicians, associated with long-term relationships between physicians and their patients, can reduce hospitalizations due to chronic diseases^43^. A study carried out in 2018 demonstrated a reduction in hospitalizations for conditions sensitive to primary care^44^, associated with the advance of the Family Health Strategy’s coverage in Brazil. On the other hand, we found a significant increase in the prevalence of hospitalizations among people with private health insurance and among those who always seek the same place when they need care, which probably represent the individuals with better access to health services^45^. Unlike the progress observed in primary care offered by the SUS, initiatives to invest in this level of care by the private sector, especially for people with chronic diseases, are still incipient in the country, which may have influenced this increase in hospitalizations. Complementarily, it is expected that there has been an increase in the diagnosis of conditions that occasionally require hospitalization, especially in people with poor health^12,22,46,47^.

Our study found variations in the prevalence of hospitalizations across Brazilian regions. In both PNS editions, the South and Midwest regions have the highest prevalence. The Southeast region, on the other hand, had the highest prevalence increase across all regions, while the North and Northeast regions remained below the national average. These findings reflect the large historical regional differences observed in Brazil, in relation to socioeconomic characteristics and social and health investments. The 2018 IBGE Household Budget Survey showed, for example, a huge regional discrepancy in health expenditures *per capita*, ranging from R$4.70 in the North region to R$70.04 in the Southeast region^48^. In addition, there are also differences in relation to age structures, HDI, provision of health services, and epidemiological profiles^22,49^.

In evaluating the estimates of hospitalizations from the two surveys, with the official number of hospitalizations, we found that the number of obstetric hospitalizations, in the public and supplementary health systems, was underestimated, as well as the number of non-obstetric hospitalizations covered by health insurance. Non-obstetric hospitalizations financed by the SUS were overestimated as well. As the information on hospital admissions referred to the last 12 months, there may have been a memory bias among the participants. In addition, the PNS items analyzed in this study refer to all of the household residents, but were answered by only one resident, which can lead to greater inaccuracy in the information. One of the limitations of household surveys is the bias associated with the use of secondary informants^50^. However, the discrepancy between the actual number of live births and the estimates of births in the two surveys may indicate the need for some methodological adjustment.

With the aim of ensuring greater homogeneity in gender distribution in the different age groups, in the calculations of the prevalence of hospitalizations, we chose not to include obstetric hospitalizations. Thus, the prevalence figures we found are lower than those previously published. This methodological decision has been observed in previous studies^13,14,51^. Nevertheless, it should be noted that this methodological decision may lead to losses in mean estimates and consequent reduction in statistical power, making it difficult to identify differences between editions. Our focus, however, is on information in which significant differences were observed.

Our study had as a limitation the absence of administrative data regarding hospitalizations by health insurance in 2013 and we used, as an alternative, data from 2014 in the comparison with the 2013 PNS, which may generate bias in the comparison. Likewise, the divergences between the administrative data and the estimates of the two PNS may represent a greater amplitude in the degree of uncertainty of our findings, resulting from under or overestimates observed in the two PNS editions. Another issue is that, due to the large sample size in both PNS editions, minimal differences can be considered statistically significant and the differences found in this work must be evaluated not only in statistical terms, but in their epidemiological significance.

The 2019 PNS identified a predominance of hospitalizations by people with chronic diseases. This population subgroup should be better studied, identifying risk factors for hospitalization. Special attention should be given to heavy users, especially in the determinants of re-hospitalization. It is also necessary to evaluate the huge number of small hospitals that exist in Brazil. The effectiveness of the hospital care network should be investigated, bringing information to support new public policies, for a more rational use of financial resources.

## CONCLUSIONS

There was an increase in overall hospitalization rates in the period between the 2013 and 2019 PNS, especially among people with better access to health services. In addition, the hospitalization profile itself has changed. While in the 2013 edition, hospitalizations of people without chronic diseases predominated in the total number of hospitalizations, in the PNS 2019 more than half of the total number of hospitalizations were of people with chronic diseases.

It was evident that changes in the demographic and epidemiological profile of the Brazilian population are already impacting hospitalization rates. The number of chronically ill people in the Brazilian population has doubled, but the prevalence of hospitalization among them has decreased, probably because they have not yet developed complications. In this context, accessible and effective Primary Health Care becomes even more relevant, otherwise more hospitalizations and higher costs can be expected in the medium term. In addition, the hospital care policy must be continuously improved, seeking efficiency and cost reduction. Without these measures, there is a serious risk of collapse of Brazil’s health systems.
