# Supplementary material for: Hospitalizations in Brazil according to National Health Survey estimates, 2013 and 2019
Source: Rev Saude Publica. 2023 Sep 29;57:73. doi: 10.11606/s1518-8787.2023057004395 (PMC10547397; doi:10.11606/s1518-8787.2023057004395)
Supplement: Translation

# Translation: Hospitalizações no Brasil pelas estimativas da Pesquisa Nacional de Saúde, 2013 e 2019

**Authors:** André Oliveira Andrade, Sandra Rêgo de Jesus, Sostenes Mistro

**Affiliations:** I Brasil Universidade Federal da Bahia. Instituto Multidisciplinar em Saúde. Programa de Pós-Graduação em Saúde Coletiva. Vitória da Conquista, BA, Brasil; II Brasil Universidade Estadual do Sudoeste da Bahia. Departamento de Ciências da Saúde. Vitória da Conquista, BA, Brasil; III Brasil Universidade Federal da Bahia. Instituto Multidisciplinar em Saúde. Vitória da Conquista, BA, Brasil

**Keywords:** Hospitalização, Hospitais, Estatísticas Hospitalares, Inquéritos Epidemiológicos, Estudos Transversais

## Abstract

**OBJETIVO:**

Comparar o perfil e a prevalência das hospitalizações no Brasil com base nas estimativas da Pesquisa Nacional de Saúde (PNS), 2013 e 2019.

**MÉTODOS:**

Estudo transversal seriado que utilizou os dados das PNS 2013 e PNS 2019. O desfecho foi ter ficado internado por 24 horas ou mais nos últimos 12 meses. Calculamos a proporção da população nas diversas categorias de faixa etária, de presença ou ausência de doenças crônicas e de percepção do estado de saúde. Estimamos o número total de hospitalizações e a proporção correspondente a cada categoria de faixa etária, de doença crônica e de percepção do estado de saúde. Calculamos a prevalência de internação segundo fatores geográficos, socioeconômicos e condições de saúde. Comparamos as estimativas das duas edições da PNS utilizando o teste t de Student para amostras independentes. Consideramos as diferenças significativas quando o valor de p foi menor que 0,01. E finalmente comparamos as estimativas de hospitalização com os dados administrativos para avaliar a consistência dos dados.

**RESULTADOS:**

Observamos que a proporção de doentes crônicos na população passou de 15,04% para 31,48%. Este grupo foi responsável por 36,76% do total de internações em 2013 e de 57,61% em 2019. A prevalência de hospitalizações aumentou significativamente entre os dois inquéritos e os incrementos foram maiores na região Sudeste e entre pessoas que possuem plano de saúde privado. Foi encontrada uma discrepância entre os dados administrativos e as estimativas dos inquéritos. As internações obstétricas e as internações por plano de saúde foram subestimadas.

**CONCLUSÃO:**

Houve um aumento nas taxas globais de hospitalização no período compreendido entre as PNS 2013 e 2019, especialmente entre as pessoas com melhor acesso aos serviços de saúde. O perfil de hospitalização também mudou – na PNS 2013 predominaram internações de pessoas sem doenças crônicas. Isto se inverteu na PNS 2019.

## INTRODUÇÃO

O hospital é o equipamento de mais alto custo em um sistema de saúde e pode representar uma fonte significativa de desperdício de recursos, a ponto de comprometer a eficiência e efetividade desse sistema^1,2^. Em 2019 os gastos com assistência hospitalar e ambulatorial corresponderam a 49,94% das despesas públicas com a saúde no Brasil, mais que o dobro do que foi gasto com a atenção primária, no mesmo ano^3^. No entanto, apesar desse grande montante de recursos, necessário à manutenção da atenção hospitalar, não se imagina prescindir da eficácia proporcionada pelo aparato tecnológico concentrado em um hospital, especialmente em condições críticas de saúde.

É provável que o gasto com a atenção hospitalar no Brasil seria proporcionalmente maior sem a expansão dos investimentos em atenção primária que se deu após a instituição do Sistema Único de Saúde (SUS) e, principalmente, após a implantação da Estratégia de Saúde da Família. A partir dessas mudanças, houve uma melhoria significativa na atenção à saúde. O local majoritário de atendimento migrou do ambulatório para o posto/centro de saúde^4^ e, gradualmente, o hospital tem deixado de ser a porta de entrada do sistema de saúde. No entanto, em 2019, mais de 25% das pessoas ainda procuraram o hospital como local de primeiro atendimento em saúde^5^.

O hospital e a atenção primária se completam dentro de um sistema de saúde. A atenção primária tem como função o cuidado integral e longitudinal à saúde do indivíduo. Em seu âmbito, são promovidas desde ações preventivas e educativas, até cuidados de reabilitação ou mesmo paliativos^6^. Por sua vez, o hospital deve resolver determinadas situações complexas, preferencialmente de modo pouco frequente. Portanto, o que se almeja é um sistema de saúde com uma atenção primária robusta e efetiva, que proporcione as menores taxas possíveis de hospitalização^7^.

As taxas de hospitalização podem ser influenciadas por fatores sociais, econômicos, comportamentais, ambientais e demográficos, os mesmos fatores que influenciam a saúde de uma população^8^. Condições como desemprego, baixa renda, restrições e ausência de envolvimento familiar são, como exemplo, preditoras de maior frequência de internação de crianças^9^. Nos Estados Unidos, 11% da variabilidade na internação hospitalar por influenza é determinada por fatores sociais^10^. Grupos que possuem piores indicadores socioeconômicos, como baixa renda e escolaridade, têm risco 2,4 vezes maior de admissão hospitalar ou têm doenças respiratórias em formas mais graves^11^. A oferta de serviços de saúde é outro fator que afeta fortemente as taxas de internação hospitalar. Castro et al.^12^ mostraram em 2005 que “quanto maior o número médio de leitos por habitantes, maior a chance de internação e quanto maior o número de médicos por habitantes, menor a chance de internação”. Da mesma forma, há ainda outros fatores associados às hospitalizações, embora fracamente, como o percentual de analfabetismo, a proporção de leitos do SUS, a urbanização e a cobertura por planos de saúde^13^.

Ainda que as taxas de hospitalização sejam influenciadas por diversas variáveis, em dimensões distintas, o principal fator associado às admissões hospitalares é a necessidade de saúde. As pessoas que se internam apresentam maior número de doenças crônicas, pior estado de saúde, pior estado funcional e mais restrições à realização de atividades habituais^14^. Da mesma forma, a prevalência de internação hospitalar em pessoas com multimorbidades é o dobro em relação às de pessoas sem multimorbidade^15^. Essas relações entre doenças crônicas, comorbidades e internações hospitalares devem produzir mudanças importantes nas hospitalizações no Brasil, uma vez que o país está passando por uma transição demográfica e epidemiológica com aumento de doenças crônicas^16^.

O perfil demográfico e epidemiológico da população influencia a demanda de hospitalização. No Brasil, desde 1980 as taxas de fecundidade e de natalidade têm diminuído ao passo que a expectativa de vida ao nascer tem aumentado^17^. Com menos nascimentos, a demanda por leitos de obstetrícia e pediatria vem diminuindo e naturalmente houve uma redução na oferta. No entanto, a prevalência de doenças crônicas aumenta com o envelhecimento da população. Porém, paradoxalmente, esta redução na oferta de leitos e diminuição na taxa de hospitalização também tem sido observada na clínica médica e na clínica cirúrgica, ainda que em menor intensidade^17^.

Em 2013, o Instituto Brasileiro de Geografia e Estatística (IBGE) em associação com o Ministério da Saúde (MS) realizou a primeira Pesquisa Nacional de Saúde (PNS). A pesquisa tinha muitos objetivos, dentre eles o de avaliar o sistema de saúde nacional no que se refere ao acesso e uso de seus serviços, assim como dimensionar o acesso à assistência médica em diferentes níveis de atenção^18^. O inquérito revelou que 53,4% dos domicílios estavam cadastrados em Unidades de Saúde da Família, que correspondem à Atenção Primária no Brasil^19^. Posteriormente, em 2019, foi realizada a segunda edição do inquérito, com alterações em algumas questões e uma ampliação do questionário^20^. Ambas as edições da PNS apresentaram informações sobre hospitalizações e, nesta segunda edição, foi observado um aumento no cadastro dos domicílios na atenção primária para 60,0%^21^.

Segundo os dados da PNS 2013, 6,0% dos indivíduos haviam ficado internados por 24 horas ou mais nos últimos 12 meses^19^, prevalência essa que aumentou para 6,6% na PNS 2019^21^. De 1999 até 2016, a taxa de hospitalizações financiadas pelo SUS vinha diminuindo gradativamente^15,17,22^. Após essa redução sustentada, em 2017 foi identificado um aumento dessa taxa em relação ao ano anterior^22^, e esta elevação também foi percebida na última edição da PNS, em 2019. Ainda não está claro se estes fatos significam pontos fora da curva ou uma nova tendência de crescimento das hospitalizações no Brasil. Nosso estudo pretendeu esclarecer esta questão. Com base nas estimativas das PNS de 2013 e de 2019, tivemos como objetivos comparar a prevalência de internação segundo fatores geográficos, socioeconômicos e condições de saúde e identificar eventuais mudanças no perfil das hospitalizações no Brasil.

## MÉTODOS

Este trabalho é um estudo transversal seriado que utilizou os dados das PNS 2013 e PNS 2019, ocorridas respectivamente entre agosto de 2013 e fevereiro de 2014^23^, e entre agosto de 2019 e março de 2020^20^, cujo objeto de estudo foi a população brasileira.

As entrevistas da PNS 2013 ocorreram em 64.348 domicílios, em cerca de 1.600 municípios de todo o Brasil^24^, com informações sobre 205.546 moradores, referente a uma projeção populacional de 199.551.444 habitantes. As da PNS 2019 ocorreram em 94.114 domicílios, referentes a 279.382 moradores e uma projeção populacional de 209.589.607 habitantes. Os dois inquéritos seguiram uma metodologia similar. Os itens foram divididos em 3 grupos. O primeiro trazia informações sobre o domicílio e sobre visitas das equipes de saúde da família ou de agentes de endemia. O segundo investigava as características gerais de cada morador, rendimento, cobertura de planos de saúde, utilização de serviços de saúde e o estado de saúde dos indivíduos. As questões do terceiro grupo fugiram ao escopo deste estudo e não foram utilizadas. O questionário, o manual do entrevistador e os microdados estão disponíveis no site do IBGE^25^.

No presente estudo, nós utilizamos como desfecho a seguinte condição: ter ficado internado por 24 horas ou mais nos últimos 12 meses. Como necessidade de saúde é o principal determinante da utilização de hospitais, descrevemos a participação de doença crônica e de má percepção do estado de saúde na população do Brasil e no total de internações. Na descrição, foi incluída também a faixa etária. A partir dos microdados das duas PNS, nós estimamos a proporção da população nas diversas categorias de faixa etária, de presença ou ausência de doenças crônicas e de percepção do estado de saúde, e o número de internações relacionados com cada categoria. Com os números totais médios estimados, e seus respectivos intervalos de confiança, foi calculado o quanto cada categoria, destas variáveis, representou, proporcionalmente, no total geral de internações, em cada edição da PNS.

Para analisar as mudanças na prevalência de hospitalização, utilizamos os fatores descritos no modelo comportamental de Andersen de uso de serviços de saúde^30^. As variáveis que utilizamos foram as seguintes: como fatores predisponentes – sexo, faixa etária, cor da pele e grau de instrução; como fatores facilitadores – moradia urbana ou rural, local habitual de atendimento, presença ou ausência de plano de saúde médico, renda domiciliar *per capita* e região da federação; e como necessidade de saúde – percepção de saúde e presença ou ausência de doença crônica. Na variável faixa etária, as idades foram agrupadas em 6 categorias: 0 a 5 anos, 6 a 17 anos, 18 a 29 anos, 30 a 39 anos, 40 a 59 anos, e 60 anos ou mais. Todas as variáveis são categóricas. As prevalências de internação hospitalar e respectivos intervalos de confiança de 95% foram calculados segundo os fatores mencionados. Nos cálculos de prevalência, foram excluídas as hospitalizações para realização de partos.

As estimativas encontradas nas duas edições da PNS foram comparadas através do teste t de Student para amostras independentes. Consideramos as diferenças significativas quando o valor de p foi menor que 0,01^31^.

A consistência dos dados dos inquéritos foi avaliada através da comparação dos dados administrativos com as estimativas das duas edições. As internações estimadas pela PNS foram destrinchadas em internações cobertas pelo SUS, internações cobertas por planos de saúde e internações obstétricas. As informações sobre nascimento em hospitais em 2013 e em 2019 foram obtidas no site do IBGE – Estatísticas do Registro Civil, através do Sistema IBGE de Recuperação Automática (SIDRA)^32^. As internações pelo SUS em 2013 e em 2019 foram obtidas no Sistema de Informações Hospitalares do SUS (SIH/SUS)^33^. As informações sobre hospitalizações cobertas por planos de saúde, parcialmente ou totalmente, foram obtidas no site da Agência Nacional de Saúde Suplementar (ANS)^34^, e se referiam ao ano de 2014 e de 2019. Não foram encontrados os dados referentes ao ano de 2013.

Por se tratar de um estudo que utilizou amostragem complexa, utilizamos para análise dos dados o software estatístico Stata®, versão SE 15.1 que, através do seu módulo survey, leva em consideração o efeito do plano de amostragem. No ajuste foram incorporados os estratos, as unidades primárias de amostragem e os pesos amostrais.

A PNS 2013 foi aprovada pelo parecer N° 328.159 da Comissão Nacional de Ética em Pesquisa. A PNS 2019 foi aprovada pelo parecer N° 3.529.376 da Comissão Nacional de Ética em Pesquisa. Todos os participantes assinaram, nas duas edições, Termo de Consentimento Livre e Esclarecido, assegurando-lhes anonimato e possibilidade de desistência a qualquer momento do estudo.

## RESULTADOS

De acordo com as estimativas dos dois inquéritos, houve mudanças no perfil demográfico e epidemiológico da população brasileira entre 2013 e 2019. Foi observada mudança significativa entre pessoas com 6 a 29 anos e nas pessoas com 40 anos ou mais. O percentual de pessoas com doenças crônicas aumentou significativamente entre as duas edições da PNS e a percepção do estado de saúde modificou-se em 4 das 5 categorias, com incremento nas categorias muito bom, regular e muito ruim. Essas informações estão detalhadas na Tabela 1.

**Tabela 1:** Distribuição proporcional (%) da população do Brasil, segundo faixa etária, presença de doença crônica e percepção do estado de saúde, conforme estimativas das PNS 2013 e PNS 2019.

Variáveis	PNS 2013 – Percentual da população (IC95%)	PNS 2019 – Percentual da população (IC95%)
Faixa etária (anos)		
0–5	7,68 (7,49–7,87)	7,44 (7,26–7,63)
6–17*	19,37 (19,07–19,68)	16,61 (16,38–16,85)
18–29*	19,36 (19,06–19,67)	17,32 (17,05–17,60)
30–39	15,44 (15,15–15,73)	15,40 (15,14–15,67)
40–59*	24,98 (24,66–25,31)	26,81 (26,51–27,11)
≥ 60	13,17 (12,83–13,52)	16,41 (16,04–16,79)
Doença crônica		
Presente*	15,04 (14,64–15,44)	31,48 (31,09–31,88)
Ausente*	84,96 (84,56–85,36)	68,52 (68,12–68,91)
Percepção do estado de saúde		
Muito bom*	14,40 (13,84–14,97)	17,90 (17,39–18,42)
Bom*	59,69 (59,11–60,28)	55,10 (54,60–55,59)
Regular*	21,56 (21,14–21,99)	22,34 (21,99–22,70)
Ruim	3,59 (3,43–3,75)	3,72 (3,60–3,85)
Muito ruim*	0,75 (0,70–0,82)	0,94 (0,86–1,02)

IC95%: intervalo de confiança de 95%; PNS: Pesquisa Nacional de Saúde.

* valor de p< 0,01 no teste t de Student para comparação de amostras independentes.

Foram observadas também mudanças no perfil de internação hospitalar. Enquanto na PNS 2013 predominaram as internações por pessoas sem doenças crônicas, na PNS 2019 foi observado um predomínio dos portadores de doenças crônicas, como encontra-se apresentado na Tabela 2.

**Tabela 2:** Participação proporcional (%) no total internações hospitalares, por faixa etária, por presença de doença crônica e por percepção do estado de saúde, conforme estimativas das PNS 2013 e PNS 2019.

Variáveis	PNS 2013 – Percentual no total de internações (IC95%)	PNS 2019 – Percentual no total de internações (IC95%)
Faixa etária (anos)		
0–5	9,59 (8,42 -10,77)	8,87 (7,80–9,95)
6–17*	9,96 (8,64–11,29)	6,70 (5,97–7,44)
18–29	15,62 (14,09–17,15)	13,86 (12,69–15,02)
30–39	15,17 (13,03–17,30)	14,75 (13,40–16,09)
40–59	24,09 (21,97–26,21)	27,47 (25,51–29,43)
≥ 60	25,56 (22,21 – 28,91)	28,35 (26,42–30,28)
Doença crônica		
Presente*	36,76 (33,33–40,19)	57,61 (54,36–60,86)
Ausente*	63,24 (58,36–68,12)	42,39 (39,91–44,86)
Percepção do estado de saúde		
Muito bom	7,73 (6,79–8,67)	9,28 (8,44–10,11)
Bom	40,07 (36,06 -44,07)	36,34 (34,13–38,56)
Regular	33,12 (30,34 -35,90)	34,47 (32,29–36,66)
Ruim	12,80 (11,30 -14,30)	13,94 (12,44–15,44)
Muito ruim	6,28 (4,54 -8,02)	5,97 (5,04–6,90)

IC95%: intervalo de confiança de 95%; PNS: Pesquisa Nacional de Saúde.

* valor de p< 0,01 no teste t de Student para comparação de amostras independentes.

Excluindo-se as internações para realização de partos, a prevalência de hospitalização aumentou significantemente de 5,32%, na PNS 2013, para 5,81%, na PNS 2019. Verificou-se aumento estatisticamente significante da prevalência, entre 2013 e 2019, na Região Sudeste, em ambos os sexos, em pessoas de cor de pele branca, em pessoas sem instrução, em pessoas com o grau de instrução médio completo, em pessoas de local de moradia urbano, em pessoas com plano de saúde, em pessoas que procuram sempre o mesmo local quando precisam de atendimento em saúde e em pessoas com rendimento domiciliar *per capita* maior que 3 e até 5 salários mínimos. Em contrapartida, reduções significativas da prevalência foram observadas em pessoas com doença crônica e em pessoas sem doença crônica, como pode ser observado na Tabela 3.

**Tabela 3:** Proporção de pessoas (%) com internação hospitalar nos últimos 12 meses, excluindo-se internações obstétricas. Pesquisa Nacional de Saúde, Brasil, 2013 e 2019.

Variáveis	PNS 2013 n = 203.883	PNS 2019 n = 277.195
	
	% (IC95%)	% (IC95%)
Geral*	5,32 (5,13–5,52)	5,81 (5,64–5,98)
Região		
Norte	5,08 (4,67–5,52)	4,68 (4,43–4,94)
Nordeste	4,92 (4,68–5,16)	5,06 (4,84–5,29)
Sudeste*	4,95 (4,58–5,36)	6,10 (5,77–6,45)
Sul	6,68 (6,24–7,15)	6,47 (6,13–6,83)
Centro-Oeste	6,53 (6,10–6,99)	6,88 (6,46–7,34)
Sexo		
Masculino*	4,95 (4,71–5,19)	5,44 (5,23–5,66)
Feminino*	5,67 (5,42–5,93)	6,15 (5,93–6,39)
Faixa etária (anos)		
0–5	7,87 (7,20–8,60)	8,32 (7,49–9,24)
6–17	2,83 (2,56–3,12)	2,63 (2,42–2,86)
18–29	3,33 (3,03–3,66)	3,47 (3,21–3,74)
30–39	4,44 (4,07–4,84)	4,61 (4,28–4,96)
40–59	5,91 (5,56–6,29)	6,31 (6,01–6,64)
≥ 60	10,23 (9,58–10,92)	10,58 (10,13–11,05)
Cor da pele		
Branca*	5,59 (5,33–5,86)	6,19 (5,94–6,44)
Preta	4,78 (4,28–5,34)	5,38 (4,97–5,83)
Amarela	5,48 (3,94–7,59)	4,92 (3,59–6,72)
Parda	5,13 (4,85–5,43)	5,56 (5,28–5,85)
Indígena	6,33 (4,39 – 9,03)	5,55 (3,91–7,83)
Grau de instrução^a^		
Sem instrução*	6,83 (6,29–7,42)	8,00 (7,37–8,68)
Fundamental incompleto	5,29 (5,01–5,60)	5,65 (5,40–5,92)
Fundamental completo	4,54 (4,05–5,07)	5,31 (4,84–5,84)
Médio incompleto	3,97 (3,39–4,65)	4,08 (3,66–4,55)
Médio completo*	4,42 (4,09–4,77)	5,23 (4,93–5,55)
Superior incompleto	4,07 (3,32–4,97)	4,92 (4,31–5,62)
Superior completo	5,51 (4,96–6,11)	5,86 (5,45–6,31)
Local de moradia		
Urbano*	5,39 (5,17–5,61)	5,91 (5,72–6,11)
Rural	4,91 (4,56–5,29)	5,20 (4,91–5,51)
Percepção da saúde		
Muito bom	3,10 (2,76–3,47)	3,28 (3,00–3,57)
Bom	3,77 (3,54–4,01)	4,12 (3,92–4,33)
Regular	8,39 (7,98–8,82)	9,07 (8,71–9,44)
Ruim	16,60 (15,34–17,96)	18,02 (16,72–19,41)
Muito ruim	27,68 (24,43–31,18)	26,66 (24,05–29,45)
Plano de saúde^b^		
Pessoas com plano de saúde*	6,45 (6,10–6,81)	7,60 (7,22–7,99)
Pessoas sem plano de saúde	4,88 (4,65–5,12)	5,18 (5,02–5,35)
Doença Crônica		
Pessoas com doença crônica*	12,20 (11,61–12,82)	10,59 (10,23–10,96)
Pessoas sem doença crônica*	4,09 (3,91–4,28)	3,61 (3,46–3,76)
Local que procura quando precisa de atendimento em saúde		
Sempre o mesmo lugar*	5,57 (5,34–5,81)	6,11 (5,92–6,31)
Procura locais diversos	4,45 (4,16–4,76)	4,82 (4,56–5,10)
Renda domiciliar *per capita*^c^		
Até ¼ salário mínimo	5,00 (4,51–5,54)	4,91 (4,55–5,30)
Mais de ¼ até ½ salário mínimo	4,71 (4,38–5,05)	4,80 (4,49–5,12)
Mais de ½ até 1 salário mínimo	5,41 (5,01–5,85)	6,02 (5,72–6,34)
Mais de 1 até 2 salários mínimos	5,41 (5,08–5,75)	5,94 (5,63–6,27)
Mais de 2 até 3 salários mínimos	5,73 (5,17–6,34)	6,11 (5,51–6,76)
Mais de 3 até 5 salários mínimos*	5,16 (4,46–5,97)	6,96 (6,28–7,71)
Mais de 5 salários mínimos	6,54 (5,79–7,38)	7,38 (6,65–8,18)

IC95%: intervalo de confiança de 95%; PNS: Pesquisa Nacional de Saúde.

* valor de p < 0,01 no teste t de Student para comparação de amostras independentes.

^a^ Na variável grau de instrução o n foi de 189.461 na PNS 2013 e de 259.408 na PNS 2019.

^b^ Houve uma mudança neste item: na PNS 2013 a questão era se tinha algum plano de saúde, médico ou odontológico; na PNS 2019 este item foi desdobrado, questionando-se sobre presença de plano de saúde médico.

^c^ Na variável renda domiciliar *per capita* o n foi de 277.023 na PNS 2019.

Ao analisarmos as internações para realização de partos, estimadas em cada inquérito, com os dados administrativos, referentes a nascidos vivos nos anos equivalentes, percebemos valores diferentes. De modo análogo, notamos diferença nas internações não obstétricas, tanto nas financiadas pelo SUS como nas financiadas por planos de saúde. Os valores são mostrados na Tabela 4.

**Tabela 4:** Comparação entre os dados administrativos e o número de internações hospitalares pelas estimativas das PNS 2013 e PNS 2019.

Tipo de internação	Estimativa PNS 2013 (IC95%)	2013 – Dados administrativos	Estimativa PNS 2019 (IC95%)	2019 – Dados administrativos
Internações obstétricas:				
SUS	1.247.725 (1.148.557–1.346.893)	2.113.466^a^	1.341.126 (1.242.042–1.440.211)	2.225.147^a^
Provavelmente SUS	7.323		1.498	
Plano de saúde	425.999 (354.489–497.508)	713.840^b^	457.670 (399.004–516.336)	648.174^c^
Pagas (e sem plano de saúde)	83.760 (48.100–119.420)		88.491 (63.307–113.674)	
Total	1.781.091 (1.662.829–1.899.354)	2.771.629^d^	1.908.288 (1.798.038–2.018.539)	2.752.386^d^
Internações não obstétricas				
SUS	10.631.713 (9.989.486–11.273.940)	9.407.371^a^	11.481.234 (11.005.629– 11.956.839)	10.128.465^a^
Provavelmente SUS	91.687 (39.810–143.565)		30.294 (–)	
Plano de saúde	4.239.450 (3.765.530–4.713.370)	6.084.261^b^	4.964.168 (4.674.907–5.253.428)	6.899.526^c^
Pagas (e sem plano de saúde)	682.632 (591.637–773.627)		754.818 (677.270–832.367)	
Total	15.939.768 (15.083.996–16.795.540)		17.409.054 (16.784.781–18.033.327)	
Total de Internações				
SUS	11.879.438 (11.210.696–12.548.180)	11.520.837^a^	12.822.361 (12.315.955–13.328.767)	12.353.612^a^
Provavelmente SUS	99.011 (47.512–150.509)		31.791 (–)	
Plano de saúde	4.665.449 (4.176.951–5.153.946)	6.798.101^b^	5.421.838 (5.111.183–5.732.493)	7.547.700^c^
Pagas (e sem plano de saúde)	766.391 (672.239–860.545)		843.309 (765.342–921.276)	
Total de Internações	17.720.859 (16.847.839–18.593.879)		19.317.343 (18.657.337–19.977.349)	

IC95%: intervalo de confiança de 95%; PNS: Pesquisa Nacional de Saúde; SUS: Sistema Único de Saúde; SIH/SUS: Sistema de Informações Hospitalares do SUS; ANS: Agência Nacional de Saúde Suplementar: IBGE: Instituto Brasileiro de Geografia e Estatística.

^a^ Produção hospitalar (SIH/SUS).

^b^ ANS – dados assistenciais do setor referentes a 2014.

^c^ ANS – dados assistenciais do setor referentes a 2019.

^d^ IBGE, Estatísticas do Registro Civil – 2.802.849 nascidos vivos em hospitais em 2013 e 2.783.409 nascidos vivos em hospitais em 2019; considerando se as gestações gemelares, estes números equivalem respectivamente a 2.771.629 e 2.752.386 partos.

## DISCUSSÃO

No presente estudo foi demonstrado um aumento na estimativa da prevalência de hospitalizações entre as edições da PNS de 2013 e 2019. Esse crescimento foi acompanhado de mudanças importantes no perfil epidemiológico e demográfico da população, em especial na duplicação do percentual de pessoas com doenças crônicas, juntamente com uma redução no percentual das pessoas com 6 a 29 e aumento entre as pessoas com 40 ou mais.

Assim como o nosso, outros estudos, com os dados das PNS 2013 e 2019, mostram que a prevalência de doenças crônicas aumentou no Brasil^35,36^. A elevação concomitante, observada na proporção de pessoas com doenças crônicas e de pessoas na faixa da meia idade, explica um achado aparentemente paradoxal. Como pode ser visto em nossos resultados, houve uma redução da prevalência de hospitalizações nas pessoas com doenças crônicas. Com um número maior de pessoas na faixa da meia-idade, há mais indivíduos portadores de doenças crônicas, mas que ainda não apresentaram complicações. Consequentemente, houve um aumento na proporção de portadores de doenças crônicas que não necessitam de hospitalização.

Apesar da prevalência de internação ter diminuído entre os portadores de doenças crônicas, como proporcionalmente eles aumentaram, e a hospitalização entre eles continua muito alta, houve um aumento na prevalência geral da população. A participação de portadores de doenças crônicas no total de internações aumentou de 36,76% para 57,61% entre as duas edições da PNS. Esses achados demonstram a influência do perfil epidemiológico e da demografia sobre as taxas de hospitalização no Brasil.

No Brasil, a transição epidemiológica é caracterizada por uma tripla carga de doenças. As doenças crônicas não transmissíveis (DCNT) coexistem com elevada incidência e prevalência de doenças infecto-parasitárias (DIP), e com causas externas^37^. Em 2019, proporcionalmente, a carga de DIP foi maior na primeira infância, a carga de causas externas foi maior em adultos jovens do sexo masculino, e a carga de DCNT aumentou com a idade^37^. Pelas estimativas da PNS 2019, em mais de 40% das internações, não havia diagnóstico de doença crônica. Se as internações refletirem a carga de doença, teremos predomínio de internações por DIP, na primeira infância, por causas externas, em jovens do sexo masculino, e por causas obstétricas em jovens do sexo feminino.

Situações emergentes também influenciam as hospitalizações. Em 2020, devido à pandemia de covid-19, houve um decréscimo de 15% na taxa de internação hospitalar, acompanhado de um aumento de 9% na letalidade intra-hospitalar, de pacientes com doenças cardiovasculares internadas pelo SUS^38^.

Um estudo sobre o perfil de internações hospitalares pelo SUS, entre 2013 e 2017, mostrou que em 24,4% das hospitalizações o paciente tinha 60 anos ou mais^39^. Estimamos que as pessoas com 60 anos ou mais responderam por 25,56% do total de internações, na PNS 2013, e por 28,35%, na PNS 2019, porém sem diferença estatisticamente significativa entre os dois inquéritos. O Brasil vem aumentando a expectativa de vida devido a reduções da mortalidade infantil e da mortalidade por doenças cardiovasculares, apesar da influência negativa da mortalidade por causas externas, que ocorre principalmente em adultos jovens do sexo masculino^40^. O aumento da expectativa de vida se deve, em parte, a melhora na assistência à saúde. O desafio é não só aumentar a expectativa de vida, mas principalmente os anos de vida com qualidade^41^.

De modo análogo encontramos que as pessoas com percepção do estado de saúde como ruim ou muito ruim correspondem a menos de 5% da população, mas são responsáveis por quase 20% do total de internações. Estas pessoas provavelmente têm uma pior saúde e já sabemos que as pessoas nesta situação são as que mais precisam de hospitais^14^. Uma atenção primária adequada e uma rede integrada de atenção à saúde podem reduzir internações, diminuir a permanência hospitalar destas pessoas, e também reduzir as reinternações^42^.

No Brasil, entre 2013 e 2019, aumentou a proporção de indivíduos que se consultaram com um médico no último ano. Este aumento foi maior entre os usuários do SUS, do que entre os portadores de plano de saúde^36^. Provavelmente isso é um reflexo da política de expansão da atenção primária, com a Estratégia de Saúde da Família.

A oferta adequada de médicos, associada a relações de longo prazo entre eles e seus pacientes pode reduzir as hospitalizações por doenças crônicas^43^. Um estudo realizado em 2018 demonstrou uma redução das internações por condições sensíveis à atenção primária^44^, associado ao avanço da cobertura da Estratégia de Saúde da Família no Brasil. Por outro lado, notamos um aumento significante na prevalência de internações entre pessoas com plano de saúde privado e entre aquelas que procuram sempre o mesmo local quando precisam de atendimento, as quais representam, provavelmente, os indivíduos com melhor acesso aos serviços de saúde^45^. Diferentemente do avanço observado na atenção primária ofertada pelo SUS, as iniciativas de investimento nesse nível de cuidado pelo setor privado, em especial aos portadores de doenças crônicas, ainda é incipiente no país, o que pode ter influenciado esse aumento nas internações. Complementarmente, é esperado que tenha havido um aumento no diagnóstico de condições que eventualmente necessitam de internações, especialmente nas pessoas com saúde debilitada^12,22,46,47^.

Nosso estudo encontrou variações na prevalência de hospitalizações entre as regiões brasileiras. Nas duas edições da PNS, as regiões Sul e Centro-Oeste apresentam a maior prevalência. A região Sudeste, por outro lado, teve o maior incremento de prevalência entre todas as regiões, enquanto as regiões Norte e Nordeste permaneceram abaixo da média nacional. Estes achados refletem as grandes diferenças regionais históricas observadas no Brasil, em relação às características socioeconômicas e aos investimentos sociais e em saúde. A Pesquisa de Orçamentos Familiares 2018 do IBGE mostrou, por exemplo, uma enorme discrepância regional nas despesas *per capita* com saúde, variando de R$ 4,70 na região Norte a R$ 70,04 na região Sudeste^48^. Além disso, existem diferenças também em relação às estruturas etárias, ao IDH, à oferta de serviços de saúde e aos perfis epidemiológicos^22,49^.

Na avaliação das estimativas de hospitalizações dos dois inquéritos, com o número oficial de internações, foi identificado nas PNS que o número de internações obstétricas, nos sistemas de saúde público e suplementar, foi subestimado, assim como o número de internações não obstétricas cobertas por plano de saúde. Também houve superestimação das internações não obstétricas financiadas pelo SUS. Como as informações sobre internações hospitalares se referiam aos últimos 12 meses, pode ter ocorrido um viés de memória entre os participantes. Além disso, os itens da PNS analisados neste estudo se referem a todos os moradores do domicílio, mas foram respondidos por apenas um morador, o que pode levar a uma maior imprecisão nas informações. Uma das limitações dos inquéritos domiciliares é o viés associado ao uso de informantes secundários^50^. Contudo, a discrepância entre o número real de nascidos vivos e as estimativas de partos dos dois inquéritos pode indicar a necessidade de algum ajuste metodológico.

Com o objetivo de garantir maior homogeneidade na distribuição dos sexos entre os diversos grupos etários, nos cálculos de prevalência das hospitalizações nós optamos por não incluir as internações obstétricas. Deste modo, as prevalências que encontramos são menores que aquelas previamente divulgadas. Essa decisão metodológica tem sido observada em estudos anteriores^13,14,51^. Apesar disso, deve-se ressaltar que essa decisão metodológica pode acarretar perdas nas estimativas médias e consequente redução do poder estatístico, dificultando a identificação de diferenças entre as edições. Contudo, nosso foco são as informações nas quais foram observadas diferenças significantes.

Nosso estudo teve como limitação a ausência dos dados administrativos referentes às internações por plano de saúde do ano de 2013 e usamos, como alternativa, os dados de 2014 na comparação com a PNS 2013, o que pode gerar um viés na comparação. Da mesma forma, as divergências entre os dados administrativos e as estimativas das duas PNS podem representar uma maior amplitude no grau de incerteza dos nossos achados, resultante de sub ou superestimavas observadas nas duas edições da PNS. Outra questão é que, devido ao grande tamanho de amostra nas duas edições da PNS, diferenças mínimas podem ser consideradas estatisticamente significativas e as diferenças encontradas neste trabalho devem ser avaliadas não apenas em termos estatísticos, mas em seu significado epidemiológico.

A PNS 2019 identificou um predomínio de internações por pessoas com doenças crônicas. Este subgrupo populacional deve ser melhor estudado, identificando os fatores de risco para hospitalização. Especial atenção deve ser dada aos grandes usuários, principalmente nos determinantes de reospitalização. É necessário, também, uma avaliação da enorme quantidade de hospitais de pequeno porte, que existem no Brasil. A eficácia da rede de atenção hospitalar deve ser investigada, trazendo informações para subsidiar novas políticas públicas, para uma utilização mais racional dos recursos financeiros.

## CONCLUSÕES

Houve um aumento nas taxas globais de hospitalização no período compreendido entre as PNS 2013 e 2019, principalmente entre as pessoas com melhor acesso aos serviços de saúde. Além disto o próprio perfil de hospitalização mudou. Enquanto na edição de 2013 predominaram, no total de hospitalizações, as internações de pessoas sem doenças crônicas, na PNS 2019 mais da metade do total das internações foi de portadores de doenças crônicas.

Ficou evidente que as alterações do perfil demográfico e epidemiológico da população brasileira já estão impactando as taxas de internação. O número de doentes crônicos, na população brasileira, dobrou, porém a prevalência de internação, entre eles, diminuiu, provavelmente porque eles ainda não desenvolveram complicações. Neste contexto, uma atenção primária à saúde acessível e eficaz passa a ser ainda mais relevante, do contrário pode-se esperar mais internações e maiores custos em médio prazo. Além disto, a política de atenção hospitalar deve ser continuamente aprimorada, buscando eficácia e redução de custos. Sem estas medidas, existe o sério risco de colapso dos sistemas de saúde do Brasil.
